# Shear Bond Strength of Orthodontic Brackets Bonded with Thermo-Cured Glass-Based Materials—An In Vitro Study

**DOI:** 10.3390/ma17133090

**Published:** 2024-06-24

**Authors:** Stipo Cvitanović, Ružica Zovko, Mirela Mabić, Sanja Jurišić, Nevenka Jelić-Knezović, Domagoj Glavina, Kristina Goršeta

**Affiliations:** 1 Health Care Center Prozor-Rama, Study of Dental Medicine, School of Medicine, University of Mostar, Mostar 88000, Bosnia and Herzegovina; stipo.cvitanovic@gmail.com; 2 Health Care Center Mostar, Study of Dental Medicine, School of Medicine, University of Mostar, Mostar 88000, Bosnia and Herzegovina; ruzicazovko71@gmail.com; 3 Faculty of Economics, University of Mostar, Mostar 88000, Bosnia and Herzegovina; mirela.mabic@gmail.com; 4 Study of Dental Medicine, School of Medicine, University of Mostar, Mostar 88000, Bosnia and Herzegovina; sanjajurisic10@gmail.com; 5 School of Medicine, University of Mostar, Mostar 88000, Bosnia and Herzegovina; nevenka.jelic@mef.sum.ba; 6 School of Dental Medicine, University of Zagreb, 10000 Zagreb, Croatia

**Keywords:** adhesive remnant index (ARI), enamel preparation technique, shear bond strength (SBS)

## Abstract

The results of orthodontic therapy largely depend, among other factors, on the preparation of the tooth enamel itself and the choice of material used to bond orthodontic brackets. The aim of this in vitro study was to determine the shear bond strength (SBS) and adhesive remnant index (ARI) score of thermo-cured glass–ionomers on different pretreated enamel, in comparison with the commonly used composite cement. Three commercially available nano-ionomer or highly viscous glass–ionomer cements (EQUIA Forte^®^ Fil, EQUIA Fil, Ketac Universal) and two types of compo-sites (Heliosit Orthodontic, ConTec Go!) were investigated in this study. The research involved two hundred human premolars. The teeth were cleaned and polished, then randomly divided into five groups according to the enamel preparation method and the type of material. The enamel was treated in three different ways: polyacrylic acid, phosphoric acid, 5% NaOCl + etching with phosphoric acid, and a control group without treatment. Glass–ionomer cement was thermo-cured with heat from a polymerization unit during setting. Statistical analysis was performed using a Chi-square test and one-way ANOVA for independent samples. Spearman’s Rho correlation coefficient was used to examine the relationship. Regardless of the material type, the results indicated that the weakest bond between the bracket and tooth enamel was found in samples without enamel pretreatment. The majority of the materials stayed on the brackets in samples without enamel preparation, according to ARI scores. The study’s findings demonstrated that the strength of the adhesion between the bracket and enamel is greatly influenced by enamel etching and glass–ionomer thermo-curing. Clinical investigations would be required to validate the outcomes.

## 1. Introduction

Demineralization around orthodontic brackets is a common problem during orthodontic therapy, especially in children and adolescents [1,2]. In residual plaque, colonies of Streptococcus mutans increase, forming white spots and causing enamel demineralization [3,4]. White spots appear as chalky white opacities on the smooth enamel surface, which can progress to cavitation upon probing. The most common site for bacterial accumulation is the junction between the adhesive and the enamel surface [5]. After completing fixed orthodontic therapy, which typically lasts about two years, demineralization can occur in up to 50% of patients [1,6]. An important factor to consider in addition to the strength of the bond by which brackets are attached to teeth is the prevention of development of initial caries lesions around brackets [7].

Finnema et al. have shown that soon after the bracket is connected with the composite to the surface of the tooth, the gradual loss of the composite bond begins [8].

Fixed orthodontic appliances, consisting of brackets, tubes, archwires, various wires, elastic bands, and excess adhesive, make oral hygiene maintenance difficult and provide retention areas for plaque accumulation on the enamel surface [5].

Therefore, agents that reduce the risk of caries and the amount of plaque and increase tooth resistance to caries are needed. In combination with bioactive glass, fluoride toothpaste aims to remineralize initial carious lesions [9]. Adding silver nanoparticles to adhesive systems for bracket cementation and using adhesive systems that release fluoride aim to minimize enamel demineralization as much as possible [10,11].

In 1972, Wilson and Kent introduced a new translucent glass–ionomer cement (GIC) for use in dentistry. This cement is a hybrid of silicate and polycarboxylate cement and could physically and chemically bond to both enamel and dentin [12]. Glass–ionomer cements are now widely used in clinical practice as fissure sealants, lining materials, and as the material of choice for cementing fixed prosthetic works and orthodontic brackets [5]. Modern highly viscous glass–ionomer cements are also used for remineralizing carious lesions and long-term restorative procedures, especially in pediatric dentistry [13]. In addition to their fluoride-releasing properties, glass–ionomer cements have been found to have weaker bond strength between the bracket and tooth compared to composite materials [14].

The adhesion of brackets to enamel should be sufficiently strong to withstand all loads during the course of therapy [15]. Most clinical orthodontic bonds require an estimated bond strength of 5.9–7.8 MPa. Some studies report the bond strength of brackets cemented with resin-based glass–ionomer cements ranging from 5.39–18.9 MPa [16].

Improvements in glass–ionomer cements for orthodontic use have led to the emergence of new resin-modified cements. Bishara and colleagues showed that with conditioned enamel and in a moist environment, light-polymerized resin-modified glass–ionomer adhesive systems exhibit comparable shear bond strength to traditional light-polymerized composite materials [17].

Glass–ionomer cements have the property of chemically bonding to both dentin and enamel, a coefficient of thermal expansion equal to hard dental tissues, with excellent compatibility and the ability for long-term fluoride release, resulting in an anti-caries effect [18].

Research has shown that fluoride deposition in plaque around the bonding area of brackets to teeth reduces the number of bacteria (Streptococcus mutans and Lactobacillus) [19]. It has been proven that bioactive glass–ionomer cements successfully prevent demineralization, but the issue of low adhesive strength values to enamel remains with conventional glass–ionomer cements [20]. The bond strength of brackets is one of the most important factors determining the ultimate success of orthodontic therapy.

Recent research has shown that the curing of glass–ionomer cements can be accelerated using an external energy source [21]. Heating can also generally improve their mechanical characteristics [22,23]. An innovative approach, consisting of thermo-curing the GIC during placement, is presented. It has been shown that thermally cured GIC sealant may provide long-term caries protection to fissures and pits without the need to maintain and reseal. According to this finding in previous studies, this technique is applied in this study.

Some authors have attempted to use ultrasonic instruments, composite polymerization lamps, and hot metal rods as energy sources to accelerate the curing process and thus improve the material’s mechanical properties [21,24,25]. Composite materials used for bracket cementation achieve good adhesion to enamel but do not prevent demineralization of the surrounding enamel, which is observed during and especially after wearing braces [1].

The advantages of glass–ionomer cements include chemical bonding to enamel and then acting as a fluoride depot, which directly impacts the reduction of demineralization. Additionally, glass–ionomer cements have significantly lower bacterial adhesion compared to composites.

The adhesion of materials to teeth changes over time due to chemical and thermal factors [26]. Modern studies have shown that heating glass–ionomer cements can reduce surface roughness and increase microhardness [22,25]. Research has confirmed that heating glass–ionomer cements with different polymerization units over a period of 60 s during the curing process increases the microhardness of glass–ionomer cements at all tested depths and may increase resistance to stronger forces [25]. Adding external energy through thermal curing as a “Command set” method during setting of glass–ionomer cements is crucial for achieving better mechanical properties [22,25,27,28,29]. Such thermal curing potentially protects glass–ionomer cement fillings from saliva contamination during the first 3–4 min after placing glass–ionomer cements [23].

The objectives of this research were: (1) to determine the effect of enamel preparation on the adhesive strength of high viscosity GICs compared to the gold standard—composite cement; (2) to determine whether heating high viscosity GICs for 60 s during the early stages of curing can cause an increase in adhesive strength that could meet the bonding values required for adequate retention of orthodontic brackets compared to the gold standard or two composite cements (light-cured and chemically cured); (3) to determine the adhesive remnant index (ARI score) on enamel after bracket removal.

## 2. Materials and Methods

### 2.1. Materials

This study investigated three commercially available nano-ionomer or highly viscous glass–ionomer cements (EQUIA Forte^®^ Fil, EQUIA Fil, Ketac Universal) and two types of composites (Heliosit Orthodontic, ConTec Go!) Glass–ionomer cements harden chemically; one of the composites is a light-polymerizing composite, and the other is a chemical-polymerizing composite. All research cements were used in encapsulated form in color A2. The materials were mixed using the CapMix^®^ device (3M ESPE, Seefeld, Germany).

### 2.2. Adhesion Strength Testing

The adhesion strength of the composite and high-viscosity glass–ionomer cements was measured using a universal material testing machine (Lrx Material Testing Machine, AMETEK Lloyd instruments Ltd., Bognor Regis, West Sussex, UK) at a crosshead speed of 1 mm/min. A 1 kN load cell was used, and the load at failure was captured electronically by the central processing unit.

### 2.3. Microscopic Surface Analysis

The adhesion–cohesion fracture ratio was determined using a light microscope (HMV–2000; Shimadzu Corporation, Tokyo, Japan) for microscopic surface analysis of all samples at 20× magnification.

### 2.4. Thermocycling

Thermocycling was performed using a thermocycling device with two baths, automatically maintained at 5 °C and 55 °C (Thermocycler THE1100, SD Mechatronic GMBH, Feldkirchen, Germany).

### 2.5. Adhesive Remnant Index (ARI)

The adhesive remnant index (ARI score) was assessed using scores ranging from 0 to 3:

- Score 0: no cement left on the enamel surface.
- Score 2: less than 50% of the cement left on the enamel.
- Score 3: more than 50% of the cement left on the enamel.
- Score 4: almost all cement left on the enamel.

### 2.6. Specimen Preparation—Protocol

The research involved two hundred human premolars. The teeth were cleaned, polished, and randomly divided into five groups based on enamel preparation method and type of material.

Material capsules were prepared according to the manufacturer’s instructions. After mixing the glass–ionomer cement (GIC) capsule, the material was placed on the bracket and cemented onto extracted intact teeth (premolars). Teeth with damage, demineralization, carious lesions, or fillings were excluded from the study. Composite material samples were prepared in the same manner according to the manufacturer’s instructions.

The glass–ionomer cement material was set “on demand” with thermo-curing for 60 s using a Bluephase G2 polymerization unit (Ivoclar Vivadent, Schaan, Liechtenstein; wavelength: 385–515 nm; power: 1200 mW/cm^2^). It was as close as possible to metal brackets. The output temperatures of the polymerization unit were determined using a digital thermocouple (TC 309; Dostmann Electronic GmbH, Wertheim am Main, Germany). Output temperature was 60 °C.

Setting “on demand” accelerates material setting by adding external energy through heating with a polymerization unit. The chemical process is accelerated by introducing external energy, in this case, heating from a polymerization unit.

### 2.7. Bracket Application

The study employed 3.1 mm metal brackets from Equilibrium 2, Dentaurum Gruppe, Ispringen, Germany. Following the application of one layer of cement and metal brackets, 150 g of pressure was applied. The samples were placed in distilled water ten minutes after bracket cementation and kept in an incubator for twenty-four hours at 37 °C. The samples were then exposed to 500 heat cycles in two baths, one at 5 °C and the other at 55 °C, to replicate material aging in the oral cavity.

Depending on the type of enamel pretreatment, the following four groups of samples were defined:

1. UE—untreated enamel.
2. H_3_PO_4_—etched with phosphoric acid.
3. 5.25% NaOCl + H_3_PO_4_—deproteinization with 5.25% NaOCl and etched with phosphoric acid.
4. PAA—conditioned with 10% polyacrylic acid.

### 2.8. Statistical Analysis

IBM SPSS Statistics, version 25.0 (IBM Corp., Armonk, NY, USA), was used for statistical data analysis. The Shapiro–Wilk test was used to test the normality of the distribution of numerical variables. Results are expressed as numbers, mean, and standard deviations. The Chi-square test (in the absence of expected frequencies Fisher’s exact test) and one-way ANOVA for independent samples were used to test the significance of differences. Spearman’s Rho correlation coefficient examined relationships. The limit of statistical significance was set at alpha = 0.05. *p* values that could not be expressed to three decimal places are shown as *p* < 0.001.

### 2.9. Hypothesis

The adhesive strength of the thermo-cured, high-viscosity glass–ionomer cement for bracket bonding demonstrates adhesion strength comparable to that achieved with orthodontic composite material.

## 3. Results

- Analysis of adhesion strength regarding the enamel preparation procedure

Among the three analyzed materials from GIC group, the lowest adhesion strength was found in samples that had no enamel treatment. Additionally, the frequency of debonding in untreated samples of Ketac Universal was higher compared to the other two materials. Conversely, the highest adhesion strength for EQUIA Forte^®^ Fil material was recorded in samples subjected to deproteinization with 5.25% sodium hypochlorite (NaOCl), followed by etching with phosphoric acid (PA). For EQUIA Fil and Ketac Universal, the highest adhesion strength was observed in samples etched with phosphoric acid (PA).

**Figure 1:**
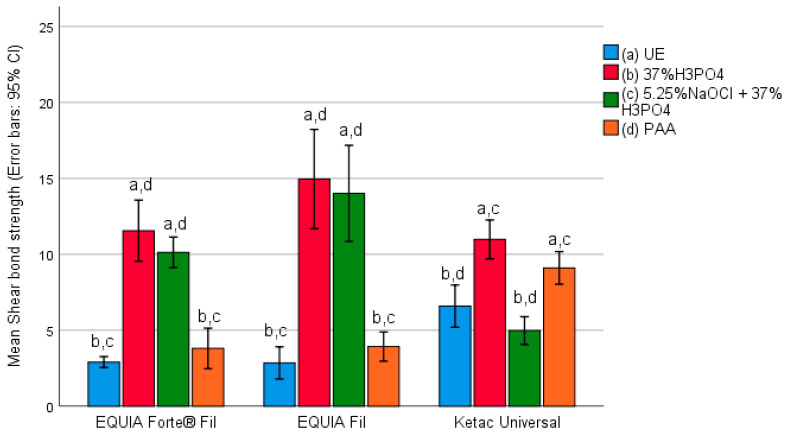
Adhesion strength of glass–ionomer cements regarding enamel preparation method.

Figure 1 shows the mean adhesion strength of the used glass–ionomer cements regarding the enamel preparation method.

In the composite group, the highest adhesion strength for the Heliosit Orthodontic material was determined in samples prepared with the deproteinization procedure using 5.25% sodium hypochlorite (NaOCl), followed by etching with phosphoric acid (PA). The lowest adhesion strength was recorded in samples without enamel preparation. For the ConTec Go! material, the highest adhesion strength was observed in samples etched with phosphoric acid (PA), while the lowest adhesion strength, similar to the Heliosit Orthodontic material, was found in samples without enamel treatment.

**Figure 2:**
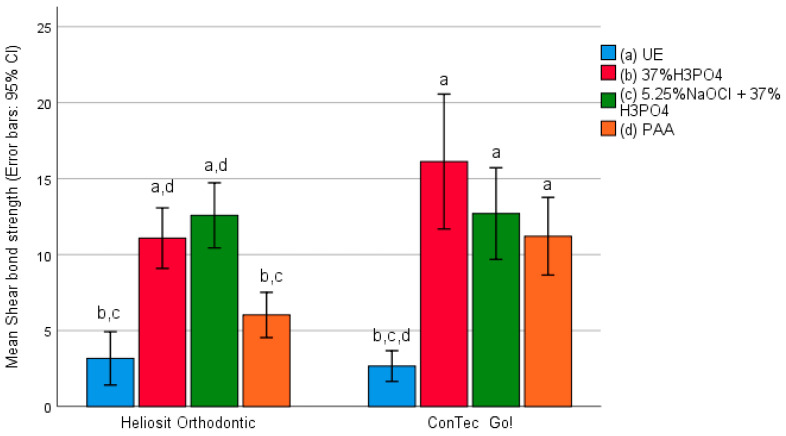
Adhesion strength of composites regarding enamel preparation method.

The mean adhesion strength of the composites used in relation to the type of enamel treatment is illustrated in Figure 2.

A statistically significant difference in adhesion strength based on the enamel preparation method was found for all three glass–ionomer cements: EQUIA Forte^®^ Fil (F = 56.228; *p* < 0.001), EQUIA Fil (F = 37.266; *p* < 0.001), and Ketac Universal (F = 26.028; *p* < 0.001).

Similarly, a statistically significant difference in adhesion strength based on the enamel preparation method was found for both composites: Heliosit Orthodontic (F = 28.330; *p* < 0.001) and ConTec Go! (F = 18.432; *p* < 0.001).

Table 1 presents the mean adhesion strengths of the materials used and the significance of differences considering the enamel preparation method.

- Analysis of the amount of residual adhesive on enamel considering the enamel preparation method

Statistically significant differences were found in the amount of residual adhesive depending on the enamel preparation method for EQUIA Forte^®^ Fil material samples (Table 2). Samples conditioned with polyacrylic acid showed a predominantly adhesive type of fracture, with a lower proportion of residual material on the enamel compared to samples prepared by other methods (Table 2). A cohesive fracture type (samples retaining all material on the enamel) was observed in samples subjected to deproteinization with 5.25% sodium hypochlorite (NaOCl) and etching with phosphoric acid (PA). All material was also retained in some samples etched with phosphoric acid (PA).

There was no statistically significant difference in the amount of residual adhesive (ARI score) regarding the enamel preparation method of the EQUIA Fil material samples (Table 2). The majority of samples of this material from all four groups, considering enamel preparation, had more than 50% residual material on the enamel (Table 2). All material on the enamel was found only in samples subjected to deproteinization with 5.25% sodium hypochlorite (NaOCl) and etching with phosphoric acid (PA).

Statistically significant differences were found in the amount of residual adhesive (ARI score) depending on the enamel preparation method of Ketac Universal material samples (Table 2). The results showed that none of the samples of this material that were not specifically prepared had residual adhesive on the enamel, while residual materials were found in samples with some form of preparation (Table 2). Additionally, more than half of the samples subjected to deproteinization with 5.25% sodium hypochlorite (NaOCl) and etching with phosphoric acid (PA) did not have residual material. The highest amount of residual material was found on samples etched with phosphoric acid (PA).

Statistically significant differences were also observed in the amount of residual adhesive (ARI score) depending on the enamel preparation method of Heliosit Orthodontic material samples (Table 2). Samples of this material that were not specially prepared had a lower proportion of residual material on the enamel compared to samples prepared using different methods (Table 2). The highest number of samples retaining all the material on the enamel was in the group etched with phosphoric acid (PA), with a significant portion also in samples subjected to deproteinization with 5.25% sodium hypochlorite (NaOCl) and etching with phosphoric acid (PA).

For ConTec Go! Material samples, statistically significant differences were found in the amount of residual adhesive (ARI score) depending on the enamel preparation method (Table 2). Samples of this material that were not specially prepared had a lower proportion of residual material on the enamel compared to those prepared using different methods (Table 2). The highest number of samples retaining all the material on the enamel was in the group etched with phosphoric acid (PA), with a significant portion also in samples subjected to deproteinization with 5.25% sodium hypochlorite (NaOCl) and etching with phosphoric acid (PA).

- Analysis of the correlation between adhesion and ARI index regarding the enamel preparation method for individual materials

Since only bond strengths of five or greater are considered adequate for the enamel-bracket bond, Table 3 presents the samples for each analyzed material with measured adhesion strength greater than five.

The obtained results showed that in both analyzed composites (Heliosit Orthodontic and ConTec Go!) and glass–ionomer cement Ketac Universal, samples meeting the reference strength value are significantly more represented, meaning their measured strength is equal to or greater than five. For the other two glass–ionomer cements, there were also slightly more samples with satisfactory strength, but they were not significantly more represented compared to samples without satisfactory strength.

After isolating samples with satisfactory strength, the association between the bond strength between enamel and bracket (adhesion) and the amount of residual adhesive (ARI index) was reanalyzed. The results are presented in Table 4.

In samples with adhesion equal to or greater than five, a significant correlation between bond strength and the amount of residual material was found. This was the case for Ketac Universal material, particularly in samples etched with phosphoric acid (PA). The correlation coefficient has a positive sign, suggesting that samples with higher strength also have a higher ARI index.

Additionally, a high, positive correlation coefficient, significant at the 0.10 level, was observed in Heliosit Orthodontic material conditioned with polyacrylic acid samples. The positive correlation coefficient implies that a higher strength is associated with a higher ARI index.

- Comparison of residual adhesive on enamel between composites and glass–ionomer cements

Significant residual adhesive (ARI index) differences between EQUIA Forte^®^ Fil material and the two analyzed composites were found for samples conditioned with polyacrylic acid: Helios (Z = −3.033; *p* = 0.002); ConTec Go! (Z = −2.805; *p* = 0.011). There was less residual adhesive on EQUIA Forte^®^ Fil samples compared to Heliosit Orthodontic and ConTec Go! samples (Table 5). A significant difference in the residual adhesive (ARI index) between EQUIA Fil and Helios materials was found for samples that were not specially prepared (Z = −3.127; *p* = 0.003), as well as for samples etched with PA (Z = −2.952; *p* = 0.009). Comparing samples of these two materials that were not specially prepared showed that EQUIA Fil samples had more residual adhesive than Helios samples (Table 5). Conversely, the analysis of samples etched with PA showed that EQUIA Fil samples had less residual adhesive than Helios samples (Table 5). Furthermore, a significant difference in the residual adhesive (ARI index) between EQUIA Fil and ConTec Go! materials was found for samples that were not specially prepared (Z = −3.574; *p* < 0.001). More residual adhesive was on EQUIA Forte^®^ Fil samples than on ConTec Go! samples (Table 5).

Comparison of Ketac Universal material with two analyzed composites revealed significant differences in the residual adhesive (ARI index) for each preparation method—samples without preparation: Heliosit Orthodontic (Z = −3.162; *p* = 0.007), ConTec Go! (Z = −2.854; *p* = 0.023); samples etched with PA: Heliosit Orthodontic (Z = −2.809; *p* = 0.007), ConTec Go! (Z = −2.296; *p* = 0.035); samples deproteinized with 5.25% NaOCl and etched with PA: Heliosit Orthodontic (Z = −3.764; *p* < 0.001), ConTec Go! (Z = −3.979; *p* < 0.001); samples conditioned with polyacrylic acid: Heliosit Orthodontic (Z = −2.571; *p* = 0.019), ConTec Go! (Z = 2.317; *p* = 0.035). For each preparation method, it was found that Ketac Universal samples had less residual adhesive than Heliosit Orthodontic samples and ConTec Go! samples (Table 5).

## 4. Discussion

Successful orthodontic therapy relies not only on a proper treatment plan but also on adequate bonding of orthodontic brackets to the enamel surface. Among the numerous factors influencing adhesion, the type of bracket and surface preparation of the teeth are crucial. The hypothesis of this research has been confirmed, as the heated high-viscosity glass–ionomer cement demonstrated adhesion strength comparable to that achieved with orthodontic composite [30].

The selection of brackets and bonding is a crucial prerequisite for conducting orthodontic treatment. The clinician must address two aspects: the mechanical properties of orthodontic brackets and the patient’s desire for short and effective treatments without discomfort.

Reduced calculus surrounding orthodontic brackets is one more benefit of the bonding technique offered by some of these adhesives, notable for their capacity to release fluoride ions. In order to stop the demineralization of the enamel surrounding the brackets, efforts are being made to develop novel materials with strong adhesion, antibacterial capabilities, and preventative qualities. A new glass–ionomer protective adhesive that has great fluoride release capabilities has recently been introduced [31].

The aim of this study was to compare the bond strength of bracket–enamel adhesion, considering the enamel treatment method, and to assess whether thermo-cured glass–ionomer cements can achieve comparable bonding to composite cements. The hypothesis was formulated that different materials produce varying bond strengths depending on the enamel preparation treatment prior to which the brackets are applied.

An adhesion, or SBS, of 5.9–7.8 MPa represents the reference value for adhesion in orthodontics. An SBS of 5.9–7.8 MPa has become the standard for adhesion in orthodontics since Reynolds introduced it in 1975 [32,33,34,35]. This value is clinically significant because the likelihood of damaging dental tissue increases by 1.3 times for each additional MPa of SBS [36]. 

Consequently, it is important to carefully adjust the bracket-to-enamel bond strength based on clinical indications. When a tooth has existing restorations or exhibits enamel abnormalities such hypoplasia, hypomineralization, or fissures, a lower bond strength may be used. This is also advisable if the treatment plan involves moving the bracket during application, as in cases of severe tooth crowding, asymmetric tooth crown forms, or persistent wear [30,37,38].

Further analysis of the bond strength of samples revealed significant differences with Heliosit Orthodontic light-polymerized material. There was a statistically significant difference between the control samples (samples without any preparation) and those for which the enamel was etched with phosphoric acid, with higher bond strength observed in samples where the enamel was etched with phosphoric acid.

The analysis of the bond strength of thermo-cured glass–ionomer cements regarding enamel pretreatment procedures has shown that glass–ionomer cements indeed justify their use in orthodontics, both in terms of bond strength and their ability to release fluoride during therapy.

For samples bonded with thermo-cured EQUIA Forte^®^ Fil glass–ionomer cement, the highest adhesion strength was found in samples where the enamel was etched with phosphoric acid. The mean adhesion strength was 11.55 MPa.

The mean adhesion strength for samples bonded with thermo-cured EQUIA Forte® Fil glass–ionomer cement, where the enamel was conditioned with 5% NaOCl and etched with phosphoric acid, was 10.19 MPa. However, these samples showed the most consistent adhesion strength. The lowest adhesion strength was 9.11 MPa, and the highest was 13.86 MPa. The mean adhesion strength for samples conditioned with polyacrylic acid was 3.8 MPa.

The mean adhesion strength for samples bonded with thermo-cured EQUIA Fil glass–ionomer cement, for which the enamel was conditioned with 5% NaOCl and etched with phosphoric acid, was 10.13 MPa. Conversely, the mean adhesion strength for samples where the enamel was conditioned with polyacrylic acid was 3.8 MPa. Flores and colleagues argued that enamel etching with phosphoric acid is a critical factor in achieving adequate adhesion when using glass–ionomer cement [39]. In our study, samples without enamel pretreatment exhibited the lowest adhesion strength, measuring at 2.85 MPa, which is insufficient to withstand the orthodontic forces during therapy. Conversely, for samples bonded with EQUIA Fil glass–ionomer cement, the highest adhesion strength was observed in samples wherein the enamel was etched with phosphoric acid with a mean adhesion strength of 11.55 MPa.

As expected, the lowest adhesion strength was found in samples without enamel treatment, with a mean adhesion strength of 2.9 MPa, which is insufficient for orthodontic forces.

A slightly lower adhesion strength of 9.1 MPa was found in samples where the enamel was conditioned with polyacrylic acid. Among these materials, higher adhesion strength was observed in samples without enamel treatment, averaging 6.57 MPa, compared to samples where enamel deproteinization with 5.25% NaOCl and etching with phosphoric acid were performed, with a mean adhesion strength of 4.98 MPa.

Among the thermo-cured glass–ionomer cements, the lowest adhesion strength was observed in samples without enamel treatment, except for Ketac Universal. The adhesion strength of untreated enamel samples of thermos-cured Ketac Universal was also higher than that of the other two materials. Conversely, the highest adhesion strength was observed in the case of thermo-cured EQUIA Forte^®^ Fil material.

A statistically significant difference in adhesion strength was found regarding the enamel preparation method for all three thermo-cured glass–ionomer cements used in the study.

The analysis of residual material/adhesive on the enamel of the analyzed composites shows that both Heliosit Orthodontic—light-polymerized—and ConTec Go!—auto polymerized—materials had residual material on all samples with different types of enamel pretreatment, while some samples that were not specifically prepared did not have adhesive residues.

The analysis of the amount of residual material on the enamel of the analyzed glass–ionomer cements shows that all samples of thermo-cured EQUIA Fil material had residual material on the enamel regardless of the preparation method. However, for thermo-cured EQUIA Forte^®^ Fil material, samples were found without residual material on the enamel—specifically, these were samples without enamel pretreatment and samples conditioned with polyacrylic acid. Results for thermo-cured Ketac Universal material showed the highest number of samples without residual material on the enamel. Residual material on the enamel was not found on any samples without specific preparation, or in more than half of the samples treated with deproteinization with 5.25% sodium hypochlorite (NaOCl) and etching with phosphoric acid (PA).

Similarly, since glass–ionomer cements contain and release fluoride and fluoride content can be replenished by local application, residual cement may not be a drawback, provided patient aesthetics are not compromised [40].

Chitnis et al. (2006) compared the in vitro shear bond strength of four adhesives: standard resin-based composite, RMGIC, giomer, and resin composite modified with polyacrylic acid. They found no statistical difference in bond strength between resin-based composite and RMGIC when the enamel surface of the resin-based composite was etched with phosphoric acid, and RMGIC with 10% polyacrylic acid; both had significantly higher mean bond strength than resin composite modified with polyacrylic acid or giomer after 1 h and 7 days. This is consistent with our study, where the highest bond strength was found in samples using composite (Heliosit Orthodontic) material, particularly in samples prepared with a deproteinization procedure using 5.25% sodium hypochlorite (NaOCl) and etching with phosphoric acid. As expected, the lowest bond strength was recorded in samples without enamel pretreatment.

In the group of samples cemented with the dual polymerization composite material (ConTec Go!), the highest bond strength was observed in the group that was etched with phosphoric acid (OPA), while other enamel pretreatment methods showed lower bond strength. Consistent with the other tested groups and materials (ConTec Go!), the lowest bond strength was found in samples without enamel pretreatment. A statistically significant difference in bond strength was found between the enamel treatment methods for both composites used in the study.

Newman et al. (2001) found that Fuji Ortho LC produced higher bond strength when enamel surfaces were treated with 10% polyacrylic acid before bonding. This finding is consistent with results obtained in this study related to Ketac Universal [41]. For Ketac Universal material samples, the highest bracket–tooth bond adhesion strength was found in samples wherein the enamel was etched with phosphoric acid, averaging 10.98 MPa.

Sfondrini et al. (2001) did not observe a significant difference in shear bond strength (SBS) between conventional composite materials and RMGIC when the enamel surface was etched with acid prior to bonding. However, under unetched conditions, the SBS achieved with RMGIC was statistically lower than that with conventional composite materials. This study used cattle permanent mandibular incisors [42].

Results from this study showed no statistically significant difference in bond strength between samples without enamel preparation compared to composite and glass–ionomer materials. Summers et al. (2004) compared the in vitro shear bond strength (SBS) and in vivo bond strength between composite materials and resin-modified glass–ionomer cements (RMGIC). For the composite group, the enamel surface was etched for 40 s with phosphoric acid, followed by a 10 s water washing, and an air-drying period before bonding. Samples for the RMGIC group were conditioned for 20 s with 10% polyacrylic acid, washed with water for 10 s, and dried with a cotton roll to eliminate extra moisture. Subsequently, Light Bond and Fuji Ortho LC were applied to the enamel surfaces and light-polymerized for 40 s. The results showed that the bond strength of Fuji Ortho LC after 30 min and 24 h was significantly lower than that of Light Bond [43].

According to the ARI analysis, the separate interfaces of RMGIC surfaces were mostly rated as score 2 (50–90% adhesive remaining on the substrate), while approximately 76% of the separate surfaces of the Transbond composite resin group were rated as score 3 (more than 90% adhesive remaining on the substrate) for 50% of the samples. These results indicate that more material remained on the substrate base when RMGIC and Transbond composite resin were used for bonding [44,45].

Our research confirmed that more residual material was present on the enamel when it was prepared with phosphoric acid. Meehan and colleagues obtained lower bond strength in groups of teeth bonded with glass–ionomer cements without enamel conditioning with 10% polyacrylic acid compared to the control group (Transbond XT) [46].

Similarly to our study, Cacciafesta and al. noted a greater increase in the bond strength of glass–ionomer cement after using phosphoric acid compared to 10% polyacrylic acid [47]. Bishara and al. found that this occurs because phosphoric acid creates a significantly rougher enamel surface, facilitating the penetration of glass–ionomer cement [48]. According to Bishara et al., as the acid concentration increases, so does the bond strength [49]. This finding aligns with the results obtained in this study.

The application of thermo-curing high-viscosity GIC in bonding brackets has not been explored in previous investigations. GICs with resin modifications have often been employed in research. Gorseta and Gavic demonstrated that thermo-curing GIC during setting can improve its mechanical properties, reduce microleakage, increase adhesion to hard dental tissues, and increase microhardness [25,28].

This study has demonstrated that thermo-cured GIC achieves adhesion strength values sufficient for good bonding and effective orthodontic therapy. The advantages of GIC over composite materials primarily include their preventive effect due to the release of fluoride ions and their antibacterial properties. Another advantage of GIC is their ability to bond to both enamel and metal surfaces. The fluoride-releasing properties of GIC have a preventive effect on enamel demineralization around brackets. Considering that orthodontic patients are at high risk for developing caries lesions, GIC could be the material of choice for bracket adhesion, especially for patients with previous caries experience.

Based on the results obtained, highly viscous GIC can be recommended for use in bracket adhesion. The procedure involves heating during setting and etching the enamel with phosphoric acid, with or without enamel deproteinization with NaOCl, depending on the material.

## 5. Conclusions

The shear bond strength values obtained verify that any of the five materials under study and enamel etching can be used to bond orthodontic brackets to enamel. The obtained results indicate that thermo-cured GIC may be effective for cementing braces. However, clinical investigations are required to validate these outcomes.

## Figures and Tables

**Table 1:** Mean adhesion strength for the materials under analysis with respect to the enamel preparation technique.

	UE (a) M (SD)	37% H_3_PO_4_ (b) M (SD)	5.25% NaOCl + H_3_PO_4_ (c) M (SD)	PAA (d) M (SD)
**Composites**				
Heliosit Orthodontic	3.17 (2.46)	11.08 (2.79)	12.58 (2.99)	6.03 ^b,c^ (2.08)
ConTec Go!	2.67 (1.42)	16.13 (6.21)	12.70 (4.21)	11.21 ^a^ (3.58)
**Glass–Ionomer Cement**				
EQUIA Forte^®^ Fil	2.90 (0.50)	11.55 (2.81)	10.13 (1.41)	3.80 ^b,c^ (1.87)
EQUIA Fil	2.85 (1.48)	14.95 (4.57)	14.01 (4.42)	3.92 ^b,c^ (1.35)
Ketac Universal	6.59 (1.95)	10.98 (1.79)	4.98 (1.27)	9.10 ^a,c^ (1.50)

M (SD)—mean (standard deviation); UE—untreated enamel; H_3_PO_4_—etched with phosphoric acid; 5.25% NaOCl + H_3_PO_4_—deproteinization with 5.25% NaOCl and etched with phosphoric acid; PAA—conditioned with 10% polyacrylic acid.

**Table 2:** The number of samples with a certain ARI score according to the enamel preparation method for each material.

	Number of Samples			
	No cement left on the enamel surface (0)	Less than 50% of the cement left on the enamel (1)	More than 50% left on the enamel (2)	Almost all cement left on the enamel (3)
**Composites**				
Heliosit Orthodontic	χ^2^ = 26.698; *p* < 0.001; Fisher’s exact test			
UE	3	6	1	0
37% H_3_PO_4_	0	0	4	6
5.25% NaOCl + H_3_PO_4_	0	1	6	3
PAA	0	4	6	0
ConTec Go!	χ^2^ = 39.650; *p* < 0.001; Fisher’s exact test			
UE	4	6	0	0
37% H_3_PO_4_	0	0	7	3
5.25% NaOCl + H_3_PO_4_	0	0	2	8
PAA	0	5	5	0
**Glass–Ionomer Cement**				
EQUIA Forte^®^ Fil	χ^2^ = 36.377; *p* < 0.001; Fisher’s exact test			
UE	1	7	2	0
37% H_3_PO_4_	0	0	7	3
5.25% NaOCl + H_3_PO_4_	0	0	4	6
PAA	3	7	0	0
EQUIA Fil	χ^2^ = 9.668; *p* = 0.052; Fisher’s exact test			
UE	0	2	8	0
37% H_3_PO_4_	0	2	8	0
5.25% NaOCl + H_3_PO_4_	0	0	6	4
PAA	0	2	8	0
Ketac Universal	χ^2^ = 23.784; *p* < 0.001; Fisher’s exact test			
UE	10	0	0	0
37% H_3_PO_4_	1	4	4	1
5.25% NaOCl + H_3_PO_4_	6	4	0	0
PAA	3	6	1	0

**Table 3:** The representation of samples for each material based on the reference adhesion strength.

	<5		≥5			
	N	%	N	%	χ^2^	*p*
**Composites**						
Heliosit Orthodontic	13	32.5	27	67.5	4.900	0.027
ConTec Go!	10	25.0	30	75.0	10.000	0.002
**Glass–Ionomer Cement**						
EQUIA Forte^®^ Fil	17	42.5	23	57.5	0.900	0.343
EQUIA Fil	16	40.0	24	60.0	1.600	0.206
Ketac Universal	8	20.0	32	80.0	14.400	<0.001

**Table 4:** Spearman’s correlation coefficient between the bond strength and ARI index of isolated samples for individual materials considering the preparation method.

	UE (a)	H_3_PO_4_ (b)	5.25% NaOCl + H_3_PO_4_ (c)	PAA (d)
**Composites**				
Heliosit Orthodontic	-	0.071	0.291	0.866
ConTec Go!	-	−0.114	0.609	0.606
**Glass–Ionomer Cement**				
EQUIA Forte^®^ Fil	-	−0.418	−0.284	—
EQUIA Fil	-	−0.087	−0.284	—
Ketac Universal	-	0.647	-	0.090

UE—untreated enamel; H_3_PO_4_—etched with phosphoric acid; 5.25% NaOCl + H_3_PO_4_—deproteinization with 5.25% NaOCl and etched with phosphoric acid; PAA—conditioned with 10% polyacrylic acid.

**Table 5:** Median of the ARI index for individual materials considering the preparation method.

	UE (a)	H_3_PO_4_ (b)	5.25% NaOCl + H_3_PO_4_ (c)	PAA (d)
**Composites**				
Heliosit Orthodontic	1	3	2	2
ConTec Go!	1	2	3	1.5
**Glass–Ionomer Cement**				
EQUIA Forte^®^ Fil	1	2	3	1
EQUIA Fil	2	2	2	2
Ketac Universal	0	1.5	0	1

UE—untreated enamel; H_3_PO_4_—etched with phosphoric acid; 5.25% NaOCl + H_3_PO_4_—deproteinization with 5.25% NaOCl and etched with phosphoric acid; PAA—conditioned with 10% polyacrylic acid.

## Data Availability

The raw data supporting the conclusions of this article will be made available by the authors on request.
